# Home-Produced Eggs as Indicators of PFAS Contamination in Food Following a Fire at a Plastic Recycling Plant

**DOI:** 10.3390/foods15101702

**Published:** 2026-05-12

**Authors:** Nina Bilandžić, Tomislav Klapec, Biljana Crevar, Ines Varga, Jelena Kaurinović, Bruno Čalopek, Maja Đokić, Damir Pavliček, Luka Cvetnić

**Affiliations:** 1 Laboratory for Residue Control, Department of Veterinary Public Health, Croatian Veterinary Institute, Savska Cesta 143, 10000 Zagreb, Croatia; varga@veinst.hr (I.V.); kaurinovic@veinst.hr (J.K.); calopek@veinst.hr (B.Č.); dokic@veinst.hr (M.Đ.); 2 Department of Applied Chemistry and Ecology, Faculty of Food Technology, Josip Juraj Strossmayer University in Osijek, F. Kuhača 18, 31000 Osijek, Croatia; tomi@ptfos.hr (T.K.); biljana.crevar@ptfos.hr (B.C.); 3 Laboratory for Microbiology and Analytical Chemistry, Department Križevci, Croatian Veterinary Institute Zagreb, Zakmardijeva 10, 48260 Križevci, Croatia; pavlicek.vzk@veinst.hr; 4 Laboratory for Mastitis and Raw Milk Quality, Department of Bacteriology and Parasitology, Croatian Veterinary Institute, Savska 143, 10000 Zagreb, Croatia; lcvetnic@veinst.hr

**Keywords:** PFAS, food, home-produced eggs, fire contamination, risk assessment

## Abstract

This study evaluated the impact of a fire at a plastic recycling plant in the suburbs of Osijek on the concentrations of per- and polyfluoroalkyl substances (PFAS) in home-produced eggs (HPE) collected from nearby settlements exposed to smoke. The assessment was conducted over three time periods following the fire. Commercial eggs from supermarkets and HPE from northwestern Croatia were also analyzed. Thirteen out of 30 compounds were quantified. In both HPE groups—the one closer to and more exposed to smoke (Zone A) and the one farther from fire (Zone B)—linear perfluorooctane sulfonic acid (L-PFOS) showed the highest detection frequency (91–100%). The highest mean concentrations of L-PFOS and the sum of the four main PFAS (∑4PFAS: PFOS, perfluorooctanoic acid (PFOA), perfluorononanoic acid (PFNA), and perfluorohexane sulfonic acid (PFHxS)), at 1.33 μg/kg, were measured in HPE from Zone A one month after the fire. In Zone B, a lower total ∑4PFAS of 0.93 μg/kg was detected. After eight months, concentrations of all quantified compounds decreased. The sums of ∑4PFAS decreased to 0.41 μg/kg (A) and 0.37 μg/kg (B), respectively. Concentrations are higher than those from northwestern Croatia and the previously determined national average. Weekly intakes of ∑4PFAS exceeded the tolerable weekly intake for toddlers and children even eight months after the fire.

## 1. Introduction

The extensive use of per- and polyfluoroalkyl substances (PFAS) in industrial and commercial products stems from their unique physicochemical properties, which result from the stable and strong carbon-fluorine bonds in both branched and linear organic chains [1]. Their combined oleophobic and hydrophobic properties enable PFAS to migrate easily through soil, water, and air via various mechanisms, including atmospheric transport on fine particles or droplets, evaporation, erosion, diffusion, advection, infiltration into groundwater, and others [1]. The high thermal and chemical stability of both short-chain and long-chain PFAS compounds prevents their degradation, making them highly persistent in the environment. This persistence may lead to bioaccumulation in plants, animals, and ultimately humans [2,3]. Perfluorooctane sulfonic acid (PFOS) and perfluorooctanoic acid (PFOA) are the two most historically used and extensively studied PFAS compounds. PFOA was primarily used in the production of fluoropolymer products (e.g., fabric, Teflon-coated plastics), while PFOS predominates in Aqueous Film Forming Foams (AFFF) and is most prevalent in industrial locations [4].

Currently, the primary point sources of PFAS pollution include fire sites where AFFF has been used, chemical plants, textile, paper, and metal coating facilities, wastewater treatment plants, and landfill leachate [5,6]. Groundwater contaminated by AFFF has been found to contain the highest mean concentrations of PFOA and PFOS [5]. Elevated PFAS concentrations have also been detected in environmental samples such as soil, water, and dust surrounding fluorochemical factories and other industrial sites [5,7,8,9,10]. In fire incidents, the hazardous chemicals present in smoke vary depending on the materials burned and the combustion conditions. These chemicals may include polycyclic aromatic hydrocarbons, dioxins, metals, formaldehyde, and others [11]. Among these are also PFAS, particularly when materials containing PFAS, such as plastic waste, are burned [11,12,13]. In commercial plastic products, PFAS may be present due to their use in processing polymers—especially fluorinated polymers—or as a result of adsorption from contaminated environments or landfills [14]. Consequently, PFAS compounds can be detected in the environment surrounding the fire scene and may be dispersed by wind into adjacent areas. Following a large fire at an industrial warehouse containing unknown chemicals and industrial waste in Melbourne, Australia, elevated concentrations of PFAS were detected in surface water and sediment samples [15]. A gradual decline in PFAS compounds in surface water was observed over several months following a large-scale industrial fire in Houston, Texas [16].

To our knowledge, there are no available studies from the literature linking the impact of fires at plastic recycling plants or other manufacturing facilities containing PFAS products to PFAS concentrations in food grown in areas exposed to the resulting smoke. Most studies have focused on the occupational exposure of firefighters to these compounds, primarily due to contact with AFFF containing PFAS [17], PFAS concentrations found in fire station dust [11,18], or PFAS in the air released during the use of fire extinguishers [19]. A study of PFAS concentrations in food grown on fire stations across South Australia revealed elevated levels of PFAS in fruits and eggs [20].

The greatest contribution to human exposure to PFAS compounds comes from food, particularly animal-based foods rich in protein, as these compounds are proteinophilic rather than lipophilic [21]. Studies have shown that albumin is the primary carrier protein for PFAS [22]. Among animal-based products, eggs exhibit the highest concentrations of these compounds [23]. Significantly higher concentrations of PFAS compounds have been measured in egg yolks compared to egg whites [24,25]. Research has also demonstrated notable differences in PFAS concentrations between commercially produced eggs—such as caged and barn eggs—and home-produced eggs (HPE). Higher concentrations have been detected in HPE obtained from hens freely roaming in private outdoor yards [26,27,28], compared to commercial eggs produced under controlled conditions [24,29]. Studies indicate regional variations in the concentration of PFOS in HPE as well as commercially produced eggs across European Union countries [28]. Significantly higher levels of PFOS and other long-chain PFAS were detected in commercial eggs from Poland [30] and Germany [31] than those found in Croatia [28]. Similarly, in HPE from Italy [26], concentrations of PFOS, PFOA, and the combined sum of the four most abundant PFAS compounds in food (∑4PFAS: PFOS, PFOA, perfluorohexane sulfonic acid (PFHxS), and perfluorononanoic acid (PFNA)) are approximately three times higher than those found in Croatia [28]. Furthermore, elevated levels of PFAS compounds have been found in HPE collected near fluorochemical plants or industrial parks in China and Belgium [24,25,32,33].

Concentrations of PFOS and PFOA are detectable in the blood of nearly all residents of developed countries due to their environmental persistence [34]. The low elimination rate and long half-life of PFAS compounds in human serum contribute to various health problems [2]. Exposure to PFASs has been linked to adverse immune effects, including a negative correlation with the immune response to routine vaccinations in children, chronic kidney disease, alterations in liver enzymes, thyroid dysfunction, elevated cholesterol levels, increased risk of hypertension, adverse reproductive and developmental outcomes, and a higher risk of kidney and testicular cancers [2,4,21,35,36]. Based on all available studies, the European Commission has established maximum levels (ML) for the four most abundant PFAS compounds in food: PFOS, PFOA, PFHxS, and perfluorononanoic acid (PFNA), as well as their combined total (∑4PFAS) in food of animal origin, such as eggs [37]. Since these compounds accumulate over time, the European Food Safety Authority (EFSA) has set a health-related exposure limit for ∑4PFAS as a tolerable weekly intake (TWI) of 4.4 ng/kg body weight (bw) [21].

The aim of this research is to assess the impact of a plastic recycling plant fire on the concentrations of PFAS compounds in eggs from free-range chickens in settlements exposed to smoke from the fire. Based on the measured PFAS concentrations, the weekly intake will be estimated, and a risk assessment will be conducted to evaluate the potential health risks to consumers.

## 2. Materials and Methods

### 2.1. Sample Collection

The fire incident occurred on 4 October 2023, and lasted for four days, at the plastic waste landfill of a plastics recycling plant located in the suburban settlement of Brijest, south of the city of Osijek, Croatia. The company’s activities include the production of plastic products and the recycling of non-metallic residues and waste, as well as the recycling of polymer PET packaging [27]. Strong, dense black smoke containing gases, soot, and particles of organic compounds spread to nearby settlements, the city of Osijek, and the wider Osijek-Baranja County area. Exposure in these settlements lasted six days, depending on the strength and direction of the wind, with a southwest wind of Beaufort force 1–3 predominating [12]. Eighteen post-fire surface soil samples collected within 10 and 20 km radii were analyzed for polycyclic aromatic hydrocarbons (PAHs), dioxins and dioxin-like polychlorinated biphenyls (PCBs), and PFAS. While low levels of dioxins and dioxin-like PCBs were detected, PFAS compounds were below the detection limit (<0.0005 mg/kg) [12].

**Figure 1:**
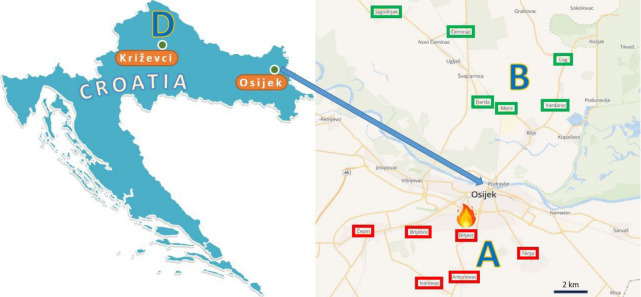
Map of study region indicating the location of egg collection after the fire in zone A and B, and northwestern Croatia (D).

HPE were collected from private households with free-range hens in settlements heavily exposed to smoke (A eggs). Fifty percent of the samples exposed to smoke originated from the villages (Brijest, Antunovac, Ivanovac, Briješće, Čepin, and Tenja), most affected due to their proximity to the plant (within a radius of 3 to 8 km) and the prevailing wind direction during the six-day exposure period (Figure 1). Eggs were collected from November 2023 to June 2024, specifically one (*n* = 39), four (*n* = 22), and eight months (*n* = 22) after the fire.

In parallel, HPE samples (*n* = 24 in November 2023; *n* = 10 in February and June 2024) were collected from settlements located further north (B eggs) of the plastics recycling plant (Mece, Darda, Čeminac, Lug, Jagodnjak, Vardarac, Kopačevo, and Švajcarnica). These settlements were situated at greater distances, within a radius of 10 to 21 km, and were less exposed to the smoke due to the wind direction.

Commercially available chicken eggs (C eggs) were purchased from supermarkets in the city of Osijek during each sampling period (*n* = 10 per period; mostly caged eggs). Each sample consisted of two eggs. The samples were stored at 4 °C until further processing.

To compare the results with other regions in Croatia, data from HPE analyses from the city of Križevci and six neighboring municipalities in northwestern Croatia (D eggs; *n* = 10) were included in the study. This area is characterized by foothill terrain with extensive agricultural production, significant forest and mineral resources, and various industrial activities, including food processing, metalworking, leather, wood, and textile industries, as well as the production of construction materials and PVC joinery. These results were incorporated into the overall presentation of PFAS concentrations in HPE across Croatia in a previous study [28].

### 2.2. Standards and Chemicals

PFAS standards were purchased from suppliers: mixture of perfluorooctanesulfonic acid (PFOS) isomers contained 79.3% linear PFOS (L-PFOS) and 20.7% branched PFOS (br-PFOS) from Cambridge Isotope Laboratories (Andover, MA, USA); pentadecafluorooctanoic acid (PFOA) from Supelco, Merck KGaA (Darmstadt, Germany); perfluorooctanesulphonamide (FOSA), capstone A, and capstone B from CPAchem (Stara Zagora, Bulgaria); perfluorononanesulfonic acid sodium salt (PFNS) from HPC Standards GmbH (Cunnersdorf, Germany). Wellington Laboratories Inc. (Guelph, ON, Canada) was the supplier for the standards: isotopically labeled standards (IS) M3HFPO-DA and MPFAC-24ES solution; sodium perfluoro-1-undecanesulfonate (PFUnDS), sodium perfluoro-1-dodecanesulfonate (PFDoDS), sodium perfluoro-1-tridecanesulfonate (PFTrDS), potassium 9-chlorohexadecafluoro-3-oxanonane-1-sulfonate (9Cl-PF3ONS), and potassium 11-chloroeicosafluoro-3-oxaundecane-1-sulfonate (11Cl-PF3OUdS) from Wellington Laboratories Inc. (Guelph, ON, Canada). Dr. Ehrenstorfer LGC Group (Teddington, UK) was the supplier for the standards: perfluorobutanoic acid (PFBA), perfluoro-n-pentanoic acid (PFPeA), perfluorohexanoic acid (PFHxA), perfluoroheptanoic acid (PFHpA), perfluorononanoic acid (PFNA), perfluoro-n-decanoic acid (PFDA), perfluoro-n-undecanoic acid (PFUnDA), perfluoro-n-dodecanoic acid (PFDoDA), perfluorotridecanoic acid (PFTrDA), perfluorotetradecanoic acid (PFTeDA), perfluorobutanesulfonic acid (PFBS), perfluoropentanesulfonic acid (PFPeS), perfluorohexanesulfonic acid (PFHxS), perfluoroheptanesulfonic acid (PFHpS), perfluorodecanesulfonic acid (PFDS), 3-H-Perfluoro-4,8-dioxanonanoic acid (DONA), perfluoro-2-propoxypropanoic acid (HFPO-DA, GenX), 1H,1H,2H,2H-Perfluorohexane sulfonic acid (4:2 FTS), 1H,1H,2H,2H-Perfluorooctane sulfonic acid (6:2 FTS), and 1H,1H,2H,2H-Perfluorodecane sulfonic acid (8:2 FTS). All analytical standards solutions had a purity of>98%.

The following chemicals were purchased from suppliers: methanol (MeOH) and acetonitrile (ACN) (ULC/MS-CC/SFC grade, ≥99.9%) from Biosolve Chimie (Dieuze, France); sodium chloride (NaCl) (analytical grade, ≥99.5%) from Supelco, Merck KGaA (Darmstadt, Germany); magnesium sulfate anhydrous (MgSO_4_) (reagent grade, ≥97%) and ammonium acetate (LC–MS grade, ≥99%) from Sigma-Aldrich (Steinheim, Germany); SPE adsorbents Bond Elut Carbon S (bulk) from Agilent Technologies (Santa Clara, CA, USA), SPE adsorbents Chromabond C18 (bulk) from Macherey-Nagel GmbH & Co. KG (Düren, Germany). A Milli-Q system (Millipore^®^, Bedford, MA, USA) was used to obtain ultrapure water (H_2_O).

Thirty PFAS analytes were prepared in methanol as standard mixtures at concentrations of 1000, 100, 10, and 1 ng/mL. A mixture of IS at 50 ng/mL in methanol was also prepared. All solutions were kept at temperatures between −20 and −10 °C.

### 2.3. Sample Extraction

After delivery to the laboratory, the egg samples were homogenized, and a portion of the homogenate was used to determine dry matter content. A vacuum dryer VS-50 SC (Kambič, Slovenia) was used for this purpose at 60 °C and 40 mbar, and the samples were dried to a constant weight. The remaining sample portion was lyophilized using a laboratory freeze dryer Alpha 2-4 LSCplus (Martin Christ Gefriertrocknungsanlagen GmbH, Osterode am Harz, Germany). The primary drying step was carried out at −40 °C and 0.250 mbar for 17 h, followed by secondary drying at 10 °C and 0.021 mbar for 60 min.

For sample preparation, 0.5 g of lyophilized egg and 1.5 g of ultrapure water were weighed into 50 mL polypropylene centrifuge tubes. The mixture was shaken using a multi-tube vortexer (VWR International GmbH, Ulm, Germany) and ultrasonicated (Grant, Grant Instruments, Cambridge, UK) for 20 min, then stored at 2–8 °C overnight. The following day, samples were equilibrated to room temperature, ultrasonicated for 10 min, shaken, and spiked with 20 µL of IS at 50 ng/mL. After spiking, the tubes were shaken again for 1 min at 2500 rpm and left in the dark for 15 min.

The eggs were extracted twice consecutively with 5 mL of acetonitrile in the same manner. The samples were first shaken at 2500 rpm for 1 min, then sonicated for 15 min in an ultrasonic bath, and centrifuged at 4000 rpm for 10 min at 20 °C (Rotanta 460R, Hettich Zentrifugen, Tuttlingen, Germany). The resulting supernatant was transferred to a clean centrifuge tube. The extraction step was repeated, and the supernatants were combined.

A slightly modified QuEChERS method from the European Union Reference Laboratory for halogenated POPs in Feed and Food was applied for sample preparation [38]. This procedure has been described in detail recently [28].

### 2.4. UHPLC-MS/MS Conditions

Quantitative analysis was carried out using a UHPLC system (1290 Infinity II) coupled to a Triple Quad LC/MS 6495C mass spectrometer with a Jet Stream Technology Ion Source (AJS ESI). The system was equipped with an InfinityLab PFC-free HPLC Conversion Kit, including an InfinityLab PFC Delay column (4.6 × 30 mm, 1200 bar), to reduce potential background contamination and interferences. Chromatographic separation was achieved on a Zorbax RRHD Eclipse Plus C18 column (2.1 × 100 mm, 1.8 µm) with a Zorbax RRHD Eclipse Plus C18 guard column (2.1 × 5 mm, 1.8 µm), maintained at 25 °C. All UHPLC-MS/MS components were supplied by Agilent Technologies (Santa Clara, CA, USA). Gradient elution was carried out at 0.3 mL/min using 2 mM ammonium acetate in H_2_O (A) and 2 mM ammonium acetate in MeOH (B). The gradient started at 25% B, followed by an increase to 60% B at 2.5 min and 80% B at 10 min. The proportion of organic phase was further increased to 90% B at 12 min and held until 14.75 min, followed by an increase to 100% B at 15 min, which was maintained until 16 min. The system was returned to initial conditions between 16 and 16.5 min and subsequently re-equilibrated until 21.5 min. The injection volume was set to 5 µL.

The mass spectrometer was operated in negative electrospray ionization (ESI) mode with dynamic multiple reaction monitoring (dMRM). The monitored transitions for analytes and IS are listed in Supplementary Table S1. The collision cell accelerator voltage (CAV) was set to 5 V for all analytes, while the fragmentor voltage was fixed at 166 V according to the manufacturer’s default settings. Source conditions were: gas temperature 250 °C, gas flow 11 L/min, nebulizer 25 psi, sheath gas temperature 375 °C, sheath gas flow 11 L/min, capillary voltage 2500 V, nozzle voltage 0 V, and EMV 250 V. Ion Funnel parameters were set at 90 V (high pressure RF) and 60 V (low pressure RF). Data processing was carried out using MassHunter Workstation software (Version 10.1).

### 2.5. Method Validation

The analytical method was validated in accordance with Commission Implementing Regulation (EU) 2022/1428 [39] and the Guidance Document provided by the European Union Reference Laboratory (EURL) [38]. The evaluated analytical parameters included specificity, selectivity, linearity, trueness, precision, limit of quantification (LOQ), and measurement uncertainty.

Selectivity and specificity were assessed through the analysis of 20 blank egg samples. Linearity was established using solvent calibration curves over seven concentration levels in the range of 0.1–20 ng/mL. Calibration curves were generated using a 1/x weighting factor, with coefficients of determination (R^2^) ≥ 0.98 for all analytes. Trueness, precision, and LOQ were evaluated by analyzing blank egg samples spiked at seven concentration levels (0.025, 0.05, 0.1, 0.15, 0.2, 0.5, and 2.5 μg/kg). Experiments were performed in duplicate at each concentration level, across six independent batches. The LOQ was defined as the lowest spiking level that met the criteria for identification, trueness, and precision. In accordance with Commission Recommendation (EU) 2022/1431, the LOQ for PFOS, PFOA, PFNA, and PFHxS in eggs was required to be ≤0.30 μg/kg [40]. Detailed validation parameters are provided in Supplementary Table S2.

### 2.6. Quality Control and Quality Assurance Procedures

Each analytical sequence consisted of a seven-point solvent calibration curve (0.1–20 ng/mL) with IS at 2 ng/mL, along with a solvent blank, a matrix blank sample, and quality control samples spiked at 0.1 and 0.2 μg/kg. Samples exceeding the calibration range were reanalyzed using an extended nine-point solvent calibration curve, including additional levels at 50 and 200 ng/mL.

Quantification was performed using a solvent calibration curve. IS was used to compensate for potential losses during sample preparation as well as for ion suppression or enhancement effects. For 11Cl-PF3OUdS, 9Cl-PF3ONS, Capstone A, Capstone B, DONA, PFDoDS, PFDS, PFHpS, PFNS, PFPeS, PFTrDA, PFTrDS, and PFUnDS, no corresponding analyte-specific IS was available. In these cases, the most suitable IS was selected based on the closest retention time or structural similarity. Apparent recoveries of quality control samples were within the acceptable range of 80–120% for compliance testing and 65–135% for monitoring purposes, in accordance with the EURL Guidance Document [38]. The overall performance of the method was additionally confirmed through successful participation in a FAPAS proficiency test (Fera Science LTD., Sand Hutton, York, UK).

### 2.7. Health Risk Assessment

Weekly intake (WI, µg/kg bw/week) was used to assess the dietary intake of PFAS, specifically the sum of four main PFAS compounds (∑4PFAS; PFOS + PFOA + PFNA + PFHxS), through egg consumption. It is calculated by multiplying the mean concentrations of ∑4PFAS (C, µg/kg) by the meal size for different age groups (MS, grams per egg portion per day), relative to body weight (g/kg bw/day) [28].

The EFSA Comprehensive European Food Consumption Database provides egg consumption data for the Croatian population by sex and age group [32]. In this study, the average chronic egg consumption (g/kg body weight/day) was used, expressed by age group as the mean and 95th percentile (P95): infants (<1 year) 1.82 and 3.99, young children (1 to 3 years) 1.66 and 4.11, other children (3 to 9 years) 1.02 and 2.81, adolescents (10 to 17 years) 0.55 and 1.57, adults (18 to 65 years) 0.48 and 1.41, and elderly (>65 years) 0.43 and 1.09. The P95 values were applied to estimate exposure for the population consuming larger quantities of eggs daily [41].

Potential risk assessment from egg consumption was conducted for HPE groups sampled in zones A and B over three time periods, as well as for group D. Eggs purchased from supermarkets (C eggs) were excluded from the evaluation because nearly all PFAS concentrations were below the LOQ values. The calculated WIs were compared with the TWI of 4.4 ng/kg bw, as defined by EFSA [21], and expressed as a percentage (%) of the TWI.

### 2.8. Statistics

Statistical analyses were conducted using Stata 13.1 for Windows (64-bit x86-64) (StataCorp LP, College Station, TX, USA). Descriptive parameters—lower bound (LB), standard deviation (SD), and range—were calculated for all PFAS compounds. For the calculation of LB values, PFAS analytes with concentrations below the LOQ are assigned a value of zero. The sum of L-PFOS and br-PFOS (∑PFOS), the sum of the four main PFAS compounds (∑4PFAS: PFOS, PFOA, PFNA, and PFHxS), and the sum of all PFAS congeners were also reported. For PFAS results measured below the LOQ, values of zero were assigned [21]. The detection frequency of each congener (%) and the contribution of each compound to the total PFAS sum were also calculated. The Shapiro–Wilk W test was used to assess data distribution. The Wilcoxon rank-sum test (Mann–Whitney U test) was used to statistically compare measured PFAS levels between HPE groups, with the significance level set at *p* < 0.05.

## 3. Results and Discussion

### 3.1. PFAS Profiles and Concentrations

In this study, the objective was to determine the concentrations of PFAS in food produced in communities surrounding a plastic recycling plant that had been exposed to smoke from a fire. Table 1 and Table 2 presents the concentrations of PFAS compounds in HPE sampled from backyards in areas with differing exposure to the fire: the area more exposed to the fire, located further south (A eggs); the less exposed area, further north of the fire (B eggs); commercial eggs from supermarkets in the city of Osijek (C eggs); and HPE sampled in northwestern Croatia (D eggs). HPE and commercial eggs were collected on three occasions: one (A1, B1, C1), four (A2, B2, C2), and eight months (A3, B3, C3) after the fire incident.

Table 1 presents the concentrations of the four main PFAS congeners—PFOS, PFNA, PFOA, and PFHxS—in HPE, along with their sum. Table 2 shows the results for other quantified congeners and the total sum of all PFAS. A total of 13 PFAS compounds were quantified, including six perfluoroalkyl sulfonic acids (PFSA: PFBS, linear PFOS (L-PFOS), branched PFOS (br-PFOS), PFHxS, PFHpS, and PFDS) and seven perfluoroalkyl carboxylic acids (PFCA: PFOA, PFNA, PFDA, PFUnDA, PFDoDA, PFTrDA, and PFTeDA). All compounds are long-chain, with PFSAs containing six or more perfluorinated carbons and PFCAs containing seven or more carbons. Among the short-chain PFAS, only the acid PFBS was detected in one sample from group A1.

The highest detection frequency in HPE groups A and B was observed for L-PFOS, ranging from 91.0% to 100%, whereas the frequency for br-PFOS ranged from 18% to 51%. Detection frequencies for PFNA, PFOA, and PFHxS were 45–90%, 5–20%, and 5–10%, respectively. In HPE samples from zones A and B, L-PFOS contributed between 72.1% and 78.4% to the total sum of ∑4PFAS (Table 1), which is higher compared to 66.7% in HPE from group D, representing the northwestern Croatia region that was not exposed to fire. In commercial eggs, L-PFOS was detected in only one sample purchased from a supermarket in Osijek one month after the fire (C1). PFAS compounds were not detected in commercial eggs purchased three and eight months after the fire (C2 and C3).

Among long-chain PFCAs (Table 2), the highest detection frequency was found for PFDA, ranging from 63.6% to 90%, and for PFDoDA, ranging from 30% to 80%. Detection frequencies for PFUnDA, PFTrDA, and PFTeDA ranged from 4.5% to 40%. Among detected PFSAs, PFHpS was found at frequencies of 4.5% to 20%, while PFDS and PFBS were detected only in HPE from zone A, at frequencies between 2.6% and 4.5%.

Regarding the total sum of all detected PFAS compounds, ∑PFOS and ∑4PFAS contributed the most. For HPE groups from zone A, their contributions ranged from 42% to 90.1% and 50.1% to 93.5%, respectively; for HPE groups from zone B, the ranges were 57.8% to 65% and 63.2% to 72.1%, respectively. In HPE group D, the contributions of ∑PFOS and ∑4PFAS to the total were 55.8% and 65.8%, respectively. Long-chain PFCAs contributed between 0.5% and 18% to the total sum of all PFAS compounds, with the highest contributions of 13.3% and 18% observed for PFDA and PFDoDA in the A1 group HPE. PFSAs contributed up to a maximum of 0.64% to the total amount. In group D, no PFSA congeners were detected, and the contribution of five long-chain PFCAs to the total ranged from 5.5% to 8%.

**Figure 2:**
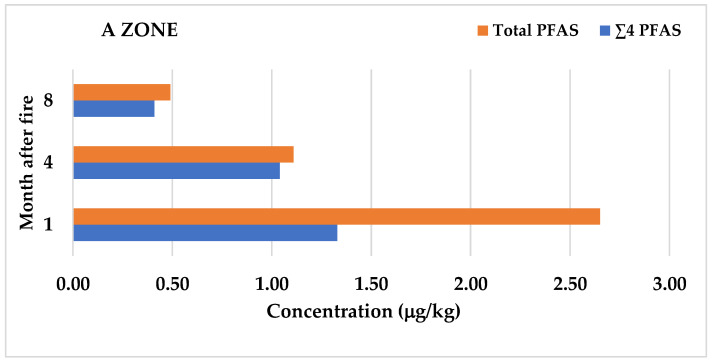

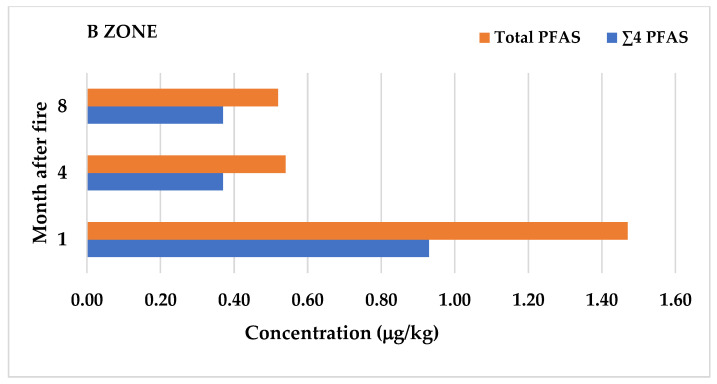
Spatiotemporal variations in ∑4PFAS and total PFAS concentrations in eggs from zones A and B following the fire.

Figure 2 highlights the spatiotemporal variations in ∑4PFAS and total PFAS concentrations in eggs from zones A and B following the fire. The highest concentrations of PFAS compounds in HPE were measured in Zone A, located south of the fire site, one month after the fire. The maximum concentration of L-PFOS reached 16.9 µg/kg, with a mean value of 0.96 µg/kg. L-PFOS levels were several times higher than those of other br-PFOS and long-chain compounds. The order of concentrations was as follows: L-PFOS > PFDoDA > PFDA > PFTeDA > br-PFOS > PFUnDA > PFNA > PFTrDA > others (< 0.06 µg/kg). In this group (A1), the highest sum of ∑4PFAS was 1.33 µg/kg, and the total sum of all quantified PFAS compounds was 2.65 µg/kg.

In the second (A2) and third (A3) measurement periods, four and eight months after the fire, respectively, a decrease in all quantified compounds was observed. L-PFOS concentrations decreased to 0.75 µg/kg and 0.32 µg/kg, respectively; the sum of ∑4PFAS declined to 1.04 µg/kg and 0.41 µg/kg; and the total sum of all PFAS compounds dropped to 1.1 µg/kg and 0.49 µg/kg. Statistical analysis revealed significant differences (*p* ≤ 0.05) in the concentrations of congeners L-PFOS, Br-PFOS, PFDoDA, and PFUnDA, as well as in the sums of ∑PFOS, ∑4 PFAS, and all PFAS compounds between groups A1, A2, and A3.

For HPE collected in Zone B, north of the fire site, lower concentrations were measured compared to Zone A. One month after the fire (B1), the highest maximum and mean values of 1.82 µg/kg and 0.70 µg/kg, respectively, were recorded for L-PFOS, followed by PFDoDA with a mean value of 0.15 µg/kg. The sum of ∑4PFAS was 0.93 µg/kg, and the total sum of all PFAS was 1.47 µg/kg. In the second (B2) and third (B3) periods, concentrations decreased further: L-PFOS values dropped to 0.29 µg/kg and 0.28 µg/kg; ∑4PFAS sums declined to 0.37 µg/kg in both periods; and total PFAS sums decreased to 0.54 µg/kg and 0.52 µg/kg, respectively. The congeners PFDS and PFBS were not detected in eggs from Zone B. Additionally, PFOA and PFHxS were not quantified in HPE from group B2.

HPE from group D consists of eggs from another region of Croatia, where no fire occurred. In these samples, the highest concentration of L-PFOS congener was 0.18 µg/kg. The sums of ∑PFOS, ∑4PFAS, and the total sum of all PFAS were determined to be 0.23 µg/kg, 0.27 µg/kg, and 0.41 µg/kg, respectively. Concentrations of all PFAS compounds in group D were lower than those in HPE from groups A and B. Significantly higher concentrations of L-PFOS, ∑PFOS, ∑PFAS, PFTrDA, PFUnDA, PFDA, and the total sum of PFAS (*p* ≤ 0.05) were observed in HPE groups A1 and B1 compared to group D. After eight months, significantly higher Br-PFOS concentrations were found in groups A3 (*p* = 0.0002) and B3 (*p* = 0.0051) relative to group D. Conversely, concentrations of PFTrDA (*p* = 0.002) and PFUnDA (*p* = 0.007) in HPE group D were significantly higher than those in group A3.

Different congeners of longer-chain perfluoroalkyl acids (PFAAs) have been quantified in eggs from various regions worldwide, reflecting both their historical and current usage [33]. PFOS is generally the most abundant congener in eggs from European countries [26,27,28,31,42,43,44], although in some countries, such as Poland, PFBA predominates [45]. Conversely, studies in China have shown that PFOA and PFBA are the most abundant compounds [24,25]. This study also confirms the highest concentrations of PFOS in chicken egg samples from areas affected by fire contamination, as well as in samples from another region of Croatia that was not exposed to fire. The greatest increase in PFOS concentrations was observed in HPE samples from settlements located south of the fire, consistent with the prevailing southwest wind direction during the event. The extensive area of the city of Osijek, including settlements from zones A and B, is topographically a flat plain with an average elevation below 100 m. The Drava River runs through the city itself. We can conclude that the topography of the area has no influence on the distribution of fire smoke, as there are no natural obstacles. Therefore, the strength and direction of the southwest wind are the most important factors affecting the distribution of smoke and contaminants. PFAS compounds are known to be distributed throughout the environment via dispersal by air currents [8].

In accordance with previous European studies, PFOS was detected at the highest concentrations in this study, contributing 72.1% to 78.4% of the total PFAS profile in HPE [27,28,33,45]. A previous study conducted in Croatia also analyzed PFAS in eggs produced using different hen farming methods [28]. Eleven PFAS compounds were quantified in HPE, with PFDS and short-chain PFBS not detected, consistent with the findings of this study. In the present study, significantly higher frequencies of detection of L-PFOS and br-PFOS in HPE (groups A and B) were observed compared to the previous Croatian study (67.6%), indicating increased contamination of eggs with PFAS compounds following the fire. Higher detection frequencies were also noted for nearly all long-chain PFAS, including PFNA, PFOA, PFDA, and PFDoDA, compared to previously reported frequencies of 43.2%, 4.05%, 43.2%, and 35.8%, respectively. The exception was PFHxS, which showed similar detection frequencies (7.43%). Previous study demonstrated low detection frequencies for all compounds in commercially produced cage eggs, with the highest frequency for L-PFOS (10.3%), comparable to the values for commercial eggs in this study. Furthermore, mean concentrations of PFAS congeners in HPE from hens kept in zones A and B affected by the fire were higher than those reported for HPE across Croatia. Even eight months after the fire, concentrations of nearly all congeners in group A3 remained elevated compared to congener levels in the previous study: Br-PFOS (0.031 µg/kg), L-PFOS (0.20 µg/kg), ∑PFOS (0.23 µg/kg), PFHxS (0.003 µg/kg), ∑4PFAS (0.26 µg/kg), and PFDA (0.035 µg/kg). However, the congeners PFNA, PFTrDA, and PFUnDA in group A3 had slightly lower concentrations than previously determined. Additionally, the obtained ∑PFOS, PFNA, and PFHxS concentrations (LB) after eight months following the fire in both A3 and B3 HPE are higher than the levels (0.27, 0.0001, and 0.0002 µg/kg, respectively) reported in the consolidated European report by EFSA [21]. In contrast, the measured PFOA values are lower than the European average of 0.106 µg/kg.

Several studies have reported PFAS contamination in HPE near fluorochemical plants [24,25,33]. These studies documented significantly higher PFAS concentrations than those found in the present study. In Belgium, PFAS contamination was detected in HPE from chickens raised near a fluorochemical plant in Antwerp [33]. Samples were collected from 35 private locations within 2, 4, and 10 km radii of this PFAS point source. Eight PFAS compounds (PFOS, PFHxS, PFBA, PFOA, PFNA, PFDA, PFUnDA, and PFDoDA) out of 17 analyzed were detected. Within the closest radius (up to 2 km from the factory), the highest detection frequencies were observed for PFOS (100%), PFOA (67%), PFBA (61%), and PFDA (39%). The highest concentrations measured were 39, 3.4, 2.8, and 0.78 µg/kg for PFOS, PFHxS, PFBA, and PFOA, respectively. At greater distances (4 and 10 km), lower PFAS concentrations were found, with PFOS at 6.5 and 4.4 µg/kg and PFOA at 0.57 µg/kg. Additionally, this study found that hens fed exclusively on kitchen food scraps produced eggs with higher PFOS and PFOA concentrations compared to hens fed only commercial feed [33].

Studies in China have reported elevated concentrations of PFAS compounds in HPE collected from backyards near fluorochemical industrial parks in Huantai County, northern China [24], and Fuxin, northwestern China [25]. Both studies demonstrate extremely high levels of PFOA and PFBA in HPE, resulting from the continuous release of these compounds into the environment. In a study conducted in Huantai County, HPE were collected within 2 km of a polytetrafluoroethylene (PTFE) production facility [24]. A total of 12 PFAS compounds were measured, with significantly higher levels found in egg yolks compared to egg whites. For example, PFOA concentrations were 368 µg/kg in yolk versus only 1.46 µg/kg in white. Very high concentrations of PFOA and PFBA, at 125 µg/kg and 22.5 µg/kg, respectively, were recorded. In contrast, significantly lower concentrations were observed for PFOS, PFNA, and PFDA, at 0.86, 0.19, and 0.26 µg/kg, respectively. The study also demonstrated significantly lower PFAS levels in commercially produced eggs, with PFBA, PFOA, and PFOS detected at concentrations of 0.85, 0.59, and 0.55 µg/kg, respectively. The findings indicate that PFAS compounds predominantly accumulate in the yolk, with PFOS retention approaching 100% and PFOA retention exceeding 90% compared to egg whites. This differential distribution pattern is suggested to result from the varying affinities of yolk and white proteins for individual PFAS compounds [24]. In a second Chinese study conducted near Fuxin City, similar findings were reported [25]. HPE samples were collected within 0.2 km of fluorochemical production plants specializing in the manufacture of perfluorobutane sulfonate (PFBS). Very high concentrations of PFBA, PFBS, and PFOA—36, 32, and 32 µg/kg, respectively—were detected. In contrast, significantly lower concentrations were observed for PFOS, PFNA, PFDA, and PFHxS, measuring 1.6, 1.4, 4.3, and 0.56 µg/kg, respectively. Both Chinese studies found that PFAS compound concentrations decrease with increasing distance from industrial plants [24,25]. The distribution of these PFAS congeners in China is attributed to the country’s current status as the world leader in the production, use, and emissions of PFOA and its salts [46]. Similarly, short-chain PFBA is now widely used in the fluorochemical industry as a primary replacement for PFOA and PFOS [20]. Consequently, in recent years, concentrations of these PFAS congeners have been more frequently detected in the environment and food in China [24,25,47].

In the context of occupational exposure to PFAS compounds, a study conducted in Australia examined their concentrations in food grown at various fire stations across South Australia [20]. The study revealed high total concentrations of PFAS (ΣPFAS), with a maximum of 1800 µg/kg at the Adelaide fire station and 927 µg/kg at the Mount Gambier fire station. The most frequently detected PFAS congeners in eggs were PFOS and PFHxS, present in 62% of the samples.

Although legislation limiting the production and spread of PFAS chemicals is in force in EU countries and other regions worldwide, a global assessment indicates an increase in PFAS contamination in the environment [1]. Soil is the main sink for PFAS compounds, and the physicochemical properties of soil play a crucial role in the bioavailability of these compounds to terrestrial organisms, particularly laying hens. Laying hens are most likely exposed to these compounds through soil scratching and dust bathing behavior, but also ingestion of soil, invertebrates, and kitchen food waste. As a result, domestic food, especially backyard eggs, is the dominant source of consumer exposure to these compounds [48].

This study highlights the contamination of food and eggs resulting from so-called point sources of PFAS compounds. Specifically, emissions of PFOS and other PFAS compounds into the atmosphere from industrial processes or disposal activities lead to pollution through sorption onto soil and water [10]. PFAS migrate from the air into the soil through two primary mechanisms: dry deposition, where ionic PFAS bind to airborne particles, and wet deposition, which occurs via precipitation such as rain and snow. Wet deposition is considered the main pathway for removing PFAS from the atmosphere and transporting them to the soil surface [49]. During the fire incident at the plastics recycling plant examined in this study, no precipitation was recorded [12], indicating that PFAS deposition occurred primarily through dry deposition. The observed decrease in PFAS concentrations in eggs over the eight months following the fire suggests that environmental contamination—specifically in the soil and subsequently in the eggs—was a singular event, with no ongoing PFAS input. This trend also implies that PFAS gradually leach into deeper soil layers and groundwater over time, as reported in the literature [50].

PFOS is the most frequently quantified PFAS in previous and this study conducted in Croatia. Since PFOS is no longer used in products within European countries, including Croatia, background concentrations likely originate from historical industrial emissions when its use was still prevalent. However, a significant source of soil contamination is its widespread application via AFFF, as well as through the application of biosolids and recycled water from wastewater treatment plants to agricultural and pasture soils [51]. PFOS exhibits high persistence and strong adsorption capacity toward soil particles, with total organic carbon content and the surface chemical properties of soils being key factors influencing PFOS sorption [48]. The sorption of PFOS into soil is further enhanced by the presence of dissolved organic carbon and other contaminants such as PFOA and Pb^2+^. Research also indicates that soil and dust serve as ultimate reservoirs for long-chain perfluorocarboxylic acids (PFCAs), such as PFUnDA and PFTrDA, in the environment [10]. The reduction in background PFAS concentrations can take years due to atmospheric transport, which distributes these molecules over long distances [1,52].

Following the gradual discontinuation of production of the two most common long-chain compounds, PFOS and PFOA, since 2002, some European countries have recorded a gradual decrease in their soil concentrations [33], as evidenced by reduced levels in the blood of the Swedish and Danish populations since 2000 [53,54].

### 3.2. Health Risk Estimation

**Figure 3:**
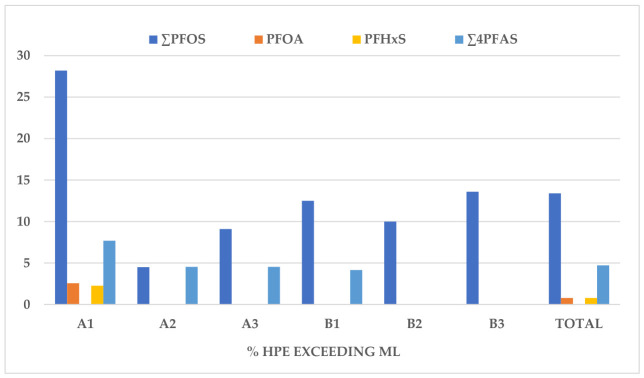
Frequency of HPE exceeding the ML in eggs from zones A and B.

The measured concentrations of PFOS, PFOA, PFNA, PFHxS, and ∑4PFAS were compared with the ML defined by Commission Regulation (EU) 2023/915 [37]. The frequency of HPE samples exceeding the ML is presented in Figure 3. The highest frequency of positive HPE was observed in group A1, with 28.2% of PFOS concentrations exceeding the ML of 1 µg/kg. Additionally, 2.56% of eggs in this group exceeded the ML for PFOA (0.30 µg/kg) and PFNA (0.70 µg/kg). Samples from zone B exhibited a lower incidence of exceedances. Lower frequencies of positives were recorded in groups A3 and B3, at 9.1% and 13.6%, respectively. The overall frequency of PFOS contamination above the ML across all collected eggs was 13.4%. No concentrations exceeding the ML were detected in group D. Furthermore, HPE samples with ∑4PFAS levels above the permitted value of 1.7 µg/kg were found at frequencies of 7.7% in A1, 4.5% in A2 and A3, and 4.16% in B1, resulting in an overall frequency of 4.72%. The overall contamination frequencies for PFOS and ∑4PFAS are significantly higher than the previously reported average frequencies of 2.12% and 1.18%, respectively, in Croatia [28].

Calculations of exposure levels—WI values for ∑4 PFAS, along with comparisons to the TWI for different age groups of the Croatian population, are presented in Table 3. The highest WI was calculated for the HPE group A1, which was sampled one month after the fire. This group showed the highest values for toddlers at 15.5 ng/kg bw, children at 9.50 ng/kg bw, adolescents at 5.12 ng/kg bw, and adults at 4.47 ng/kg bw. Compared with the health limit TWI of 4.4 ng/kg bw, it is alarming that the obtained WI was 3.5 orders of magnitude higher for toddlers (351% TWI), 2.16 times higher for children, 1.16 times higher for adolescents, and 1.02 times higher for adults. The risk of exposure is even greater for consumers who consume larger weekly quantities of eggs, as calculated using the P95 value. For toddlers, exposure reaches as high as 870% TWI, while for adults it is 300% TWI.

The weekly intake of ∑4PFAS was below 4.4 ng/kg bw for adolescents and adults in group A2, as well as for children in group A3. However, significantly elevated exposures of 275% and 169% were observed for children aged 1 to 3 years and up to 10 years, respectively, based on HPE samples collected three months after the fire (group A2). High exposure levels of 91% and 79.1% were also recorded for adolescents and adults, respectively. Eight months after the fire, lower exposure levels were found in eggs sampled from zone A (group A3). The highest contribution at this time was 108% for toddlers, while other age groups showed lower values: 66.5% for children, 35.8% for adolescents, and 31.3% for adults.

For HPE from B zone, WI values above the TWI—10.8 ng/kg bw for toddlers and 6.64 ng/kg bw for children—were determined, corresponding to exposures of 246% and 151%, respectively. Lower exposures of 81.4% and 71% were observed for adolescents and adults, compared to eggs from group A1. For groups B2 and B3, the same mean values were determined, resulting in similar contributions to TWI values, with the highest exposure of 97.7% for toddlers and the lowest of 28.3% for adults.

For consumers who consume larger quantities of eggs on a weekly basis, only adults who consumed eggs sampled after eight months from group A3, as well as adolescents and adults who consumed eggs from groups B2 and B3, had exposures relative to the TWI of less than 100%.

The results indicate a significant risk for consumers of eggs from hens exposed to PFAS contamination following the fire. This conclusion is supported by a comparison with eggs from another region of Croatia (D eggs). For these eggs, exposure levels were 71.3% for toddlers and 20.6% for adults. Among consumers with higher egg intake (P95) on a weekly basis, exposure exceeded 100% only for toddlers and children, reaching 177% and 120.7%, respectively.

Comparing the obtained risk assessment results with previous values for HPE across Croatia, it is evident that the WI values determined for the HPE groups sampled eight months after the fire are significantly higher [28]. The WI for HPE from groups A3 and B3 exceeds the previous values for all age groups, which were 3.06 ng/kg bw for toddlers, 1.88 ng/kg bw for children, 0.89 ng/kg bw for adolescents, and 0.78 ng/kg bw for adults. In contrast, the WI values for group D are similar to the previously determined average Croatian values.

Very high WI for ∑4 PFAS were found in HPE sampled near fluorochemical plants in Belgium [33]. WI values as high as 208 ng/kg bw and 154 ng/kg bw were determined for toddlers and children when consuming HPE from chickens that were kept within a radius of up to 2 km from the fluorochemical plant. Even at a distance of 4 to 10 km, hens produced eggs contaminated with high PFAS values that exceeded the WI (25 ng/kg bw for toddlers and 18 ng/kg bw for children) determined in this study. Studies have shown that toddlers and children have a higher relative intake of PFAS compared to adults due to their smaller body mass and are therefore the most exposed [23,28,33]. The established exceedances of TWI values in this study are particularly concerning because the same population may be exposed to additional sources of PFAS compounds through the consumption of other foods, especially meat, offal, fish, and vegetables grown in the same gardens or backyards.

## 4. Conclusions

This study monitored the levels of PFAS compounds in HPE following a fire at a plastic recycling plant. The results indicate that HPE produced in settlements exposed to fire smoke remained contaminated with PFAS compounds for an extended period—at least eight months after the fire. Contamination affected HPE within a wide radius, extending more than 20 km from the fire site. These findings suggest that the broader area of egg production, as well as other food sources, should be monitored for contamination over a prolonged period. This recommendation is supported by the observation that even eight months post-fire, the total concentration of ∑4PFAS did not fall below the previously established average levels for HPE in Croatia.

The results indicate a predominant frequency of HPE contamination with long-chain PFAS, consistent with the scientific literature. L-PFOS was detected most frequently across all HPE groups; specifically, the combined sum of L-PFOS and Br-PFOS contributed the most to the total ∑4PFAS concentration.

The determined concentrations indicate that eggs may significantly contribute to the population’s exposure to the sum of ∑4PFAS, which, over the long term, can lead to serious health problems. Protecting the most vulnerable groups, such as toddlers and small children, should be a priority.

The results of this study highlight the importance of determining PFAS levels in HPE, as well as in other foods grown outdoors near fires or pollution epicenters. Such events must be accompanied by rigorous monitoring of food in the period following the incident, and appropriate educational activities. To ensure a comprehensive risk assessment of human exposure in contaminated areas, all possible exposure routes should be considered, including a wide variety of food and water sources, as well as other exposure pathways such as dermal contact and inhalation.

A limitation of this study is the lack of direct evidence for the deposition pathway or transfer route to the eggs. Additionally, the study does not consider potential alternative sources of pollution. Furthermore, there is a lack of detailed environmental impact measurements, particularly comprehensive soil analyses. Future research should include an analysis of the laying hens’ diet and account for possible random variability.

## Figures and Tables

**Table 1:** Concentrations (ng/g wet weight) of four main PFAS and their sum (∑4PFAS) in HPE from laying hens in the area south of the fire zone (A), the area north of the fire (B), commercial eggs from supermarkets in the city of Osijek (C) collected at one month (A1, B1, C1), four months (A2, B2, C2), and eight months after fire (A3, B3, C3), and eggs collected in northwestern Croatia (D).

HPE Group	Statistics	Br-PFOS	L-PFOS	∑PFOS	PFNA	PFOA	PFHxS	∑4 PFAS
A1 *n* = 39	Mean ± SD	0.16 ± 0.39 ^a,b^	0.96 ± 2.68 ^a,b^	1.11 ± 3.06 ^a^	0.15 ± 0.75	0.056 ± 0.31	0.0035 ± 0.015	1.33 ± 4.12 ^a,b^
	Range	0.0–2.36	0.0–16.9	0.0–19.2	0.0–4.71	0.0–1.95	0.0–0.079	0.0–26.0
	*n* > LOQ	20	38	38	28	3	2	38
	DF (%)	51	97	97	72	8	5	97
	CTS (%)	5.9	36.1	42	5.9	2.1	0.1	50.1
A2 *n* = 22	Mean ± SD	0.25 ± 1.06 ^a^	0.75 ± 2.72 ^a^	0.99 ± 3.79 ^a^	0.029 ± 0.024	0.0038 ± 0.018	0.0049 ± 0.023	1.04 ± 3.84 ^a^
	Range	0.0–5.00	0.021–12.9	0.021–17.9	0.0–0.097	0.0–0.085	0.0–0.11	0.021–18.2
	*n* > LOQ	4	22	22	15	1	1	22
	DF (%)	18	100	100	68	5	5	100
	CTS (%)	22.6	67.5	90.1	2.6	0.3	0.4	93.5
A3 *n* = 22	Mean ± SD	0.066 ± 0.20 ^a^	0.32 ± 0.57 ^a,c^	0.38 ± 0.77 ^a^	0.022 ± 0.029	0.0039 ± 0.019	0.0022 ± 0.01	0.41 ± 0.81 ^a^
	Range	0.0–0.90	0.0–2.70	0.0–3.60	0.0–0.078	0.0–0.087	0.0–0.049	0.0–3.79
	*n* > LOQ	4	20	20	10	1	1	20
	DF (%)	18	91	91	45	5	5	91
	CTS (%)	13.6	64.4	78	4.4	0.8	0.4	83.7
B1 *n* = 24	Mean ± SD	0.15 ± 0.23 ^b^	0.70 ± 0.59 ^b^	0.85 ± 0.80	0.058 ± 0.045	0.015 ± 0.033	0.0059 ± 0.019	0.93 ± 0.87 ^b^
	Range	0.0–0.73	0.040–1.82	0.040–2.55	0.0–0.13	0.0–0.093	0.0–0.060	0.040–2.83
	*n* > LOQ	5	10	10	8	2	1	10
	DF (%)	50	100	100	80	20	10	100
	CTS (%)	10.1	47.7	57.8	4	1	0.4	63.2
B2 *n* = 10	Mean ± SD	0.044 ± 0.11	0.29 ± 0.29	0.34 ± 0.39	0.038 ± 0.021			0.37 ± 0.41
	Range	0.0–0.33	0.049–1.02	0.049–1.35	0.0–0.079			0.057–1.43
	*n* > LOQ	2	10	10	9	nd	nd	10
	DF (%)	20	100	100	90			100
	CTS (%)	8.1	53.5	61.6	7.1			68.7
B3 *n* = 10	Mean ± SD	0.057 ± 0.15	0.28 ± 0.26 ^d^	0.34 ± 0.41	0.031 ± 0.025	0.0056 ± 0.018		0.37 ± 0.42
	Range	0.0–0.47	0.077–0.99	0.077–1.45	0.0–0.068	0.0–0.056		0.077–1.52
	*n* > LOQ	2	10	10	7	1	nd	10
	DF (%)	20	100	100	70	10		100
	CTS (%)	11	54	65	5.9	1.1		72.1
C1 *n* = 10	Mean ± SD		0.0021 ± 0.0065	0.0021 ± 0.0065				0.0021 ± 0.0065
	Range		0.0–0.021	0.0–0.021				0.0–0.021
	*n* > LOQ	nd	1	1	nd	nd	nd	1
	DF (%)		10	10				10
	CTS (%)		100	100				100
C2/C3 *n* = 10	*n* > LOQ	nd	nd	nd	nd	nd	nd	nd
D *n* = 20	Mean ± SD	0.049 ± 0.036	0.18 ± 0.16 ^c,d^	0.23 ± 0.19	0.034 ± 0.022	0.005 ± 0.012	0.0014 ± 0.0061	0.27 ± 0.22
	Range	0.011–0.13	0.022–0.58	0.033–0.68	0.0–0.074	0.0–0.039	0.0–0.028	0.033–0.74
	*n* > LOQ	20	20	20	16	3	1	20
	DF (%)	100	100	100	80	15	5	100
	CTS (%)	11.9	43.9	55.8	8.4	1.2	0.3	65.8

DF (%)—detection frequency; CTS (%)—contribution to the total sum of PFAS; Significant differences (*p* ≤ 0.05) in PFAS levels between the groups: ^a^ L-PFOS, Br-PFOS, ∑PFOS, and ∑4 PFAS between A1, A2, and A3; ^b^ L-PFOS, ∑PFOS, ∑PFAS, between A1 and B1; ^c^ Br-PFOS between A3 and D; ^d^ Br-PFOS between B3 and D; nd—not detected.

**Table 2:** Concentrations (ng/g wet weight) of other eight quantified PFAS congeners and the total sum of all PFAS in home-produced eggs from laying hens in the area south of the fire zone (A), the area north of the fire (B), commercial eggs from supermarkets in the city of Osijek (C), collected at one month (A1, B1, C1), four months (A2, B2, C2), and eight months after fire (A3, B3, C3), and eggs collected in northwestern Croatia (D).

HPE Group	Statistics	PFDA	PFUnDA	PFDoDA	PFTrDA	PFTeDA	PFHpS	PFDS	PFBS	Total PFAS
A1 *n* = 39	Mean ± SD	0.35 ± 1.88 ^b^	0.16 ± 0.87 ^a,b^	0.48 ± 2.69 ^a^	0.13 ± 0.71 ^b^	0.19 ± 1.12	0.0055 ± 0.023		0.0009 ± 0.0053	2.65 ± 11.4 ^a,b^
	Range	0.0–11.8	0.0–5.45	0.0–16.9	0.0–4.46	0.0–7.01	0.0–0.14		0.0–0.033	0.0–71.7
	*n* > LOQ	30	13	27	5	3	3	nd	1	38
	DF (%)	77	33.3	69.2	12.8	7.7	7.7		2.6	97.4
	CTS (%)	13.3	6.19	18	5	7.2	0.21		0.03	100
A2 *n* = 22	Mean ± SD	0.040 ± 0.032	0.0056 ± 0.018 ^a^	0.019 ± 0.027 ^a^			0.0070 ± 0.033	0.0014 ± 0.0064		1.11 ± 3.92 ^a^
	Range	0.0–0.11	0.0–0.067	0.0–0.088			0.0–0.16	0.0–0.030		0.021–18.6
	*n* > LOQ	17	2	9	nd	nd	1	1	nd	22
	DF (%)	77.3	9.1	40.9			4.5	4.5		100
	CTS (%)	3.57	0.5	1.71			0.64	0.12		100
A3 *n* = 22	Mean ± SD	0.035 ± 0.039	0.011 ± 0.028 ^a,c^	0.022 ± 0.037 ^a^	0.0057 ± 0.027 ^c^		0.0020 ± 0.0093	0.0039 ± 0.018		0.49 ± 0.88 ^a^
	Range	0.0–0.13	0.0–0.089	0.0–0.13	0.0–0.13		0.0–0.044	0.0–0.086		0.0–3.97
	*n* > LOQ	14	3	8	1	nd	1	1	nd	20
	DF (%)	63.6	13.6	36.4	4.5		4.5	4.5		90.9
	CTS (%)	7.2	2.2	4.5	1.2		0.41	0.8		100
B1 *n* = 24	Mean ± SD	0.093 ± 0.097 ^b^	0.068 ± 0.10 ^b^	0.15 ± 0.2417	0.099 ± 0.19 ^b^	0.12 ± 0.23	0.0068 ± 0.015			1.47 ± 1.62 ^b^
	Range	0.0–0.26	0.0–0.28	0.0–0.77	0.0–0.60	0.0–0.64	0.0–0.040			0.040–5.43
	*n* > LOQ	8	4	8	3	3	2	nd	nd	10
	DF (%)	80	40	80	30	30	20			100
	CTS (%)	6.3	4.7	10.2	6.7	8.4	0.5			100
B2 *n* = 10	Mean ± SD	0.049 ± 0.040	0.021 ± 0.047	0.045 ± 0.11	0.018 ± 0.058	0.034 ± 0.11	0.0028 ± 0.0089			0.54 ± 0.76
	Range	0.0–0.14	0.0–0.14	0.0–0.35	0.0–0.18	0.0–0.34	0.0–0.028			0.057–2.61
	*n* > LOQ	9	2	3	1	1	1	nd	nd	10
	DF (%)	90	20	30	10	10	10			100
	CTS (%)	9.1	3.8	8.3	3.3	6.3	0.52			100
B3 *n* = 10	Mean ± SD	0.040 ± 0.031	0.014 ± 0.0313	0.037 ± 0.068	0.023 ± 0.071	0.031 ± 0.097				0.52 ± 0.69
	Range	0.0–0.092	0.0–0.088	0.0–0.21	0.0–0.23	0.0–0.31				0.077–2.44
	*n* > LOQ	8	2	4	1	1	nd	nd	nd	10
	DF (%)	80	20	40	10	10				100
	CTS (%)	7.8	2.8	7.1	4.4	5.9				100
C1 *n* = 10	Mean ± SD									0.0021 ± 0.0065
	Range									0.0–0.021
	*n* > LOQ	nd	nd	nd	nd	nd	nd	nd	nd	1
	DF (%)									10
	CTS (%)									100
C2/C3 *n* = 10	*n* > LOQ	nd	nd	nd	nd	nd	nd	nd	nd	nd
D *n* = 20	Mean ± SD	0.033 ± 0.023	0.027 ± 0.022 ^c^	0.031 ± 0.029	0.023 ± 0.025 ^c^	0.027 ± 0.043				0.41 ± 0.34
	Range	0.0–0.071	0.0–0.059	0.0–0.10	0.0–0.060	0.0–0.16				0.033–1.13
	*n* > LOQ	15	13	13	10	8	nd	nd	nd	20
	DF (%)	75	65	65	50	40				100
	CTS (%)	8.00	6.6	7.6	5.5	6.5				100

DF (%)—detection frequency; CTS (%)—contribution to the total sum of PFAS; Significant differences (*p* ≤ 0.05) in PFAS levels between the groups: ^a^ PFDoDA, PFUnDA, and total PFAS between A1, A2, and A3; ^b^ PFDA, PFUnDA, PFTrDA, and total PFAS between A1 and B1; ^c^ PFUnDA and PFTrDA between A3 and D; nd—not detected.

**Table 3:** Weekly intakes for the sum ∑4PFAS and risk characterization in different age groups of the Croatian population for HPE from settlements heavily (A eggs) and less exposed to smoke (B eggs), and HPE collected in northwestern Croatia (D eggs).

	Weekly Intake (ng/kg bw)															
HPE Group	Toddlers				Children				Adolescents				Adults			
	Mean		P95		Mean		P95		Mean		P95		Mean		P95	
	WI	TWI%	WI	TWI%	WI	TWI%	WI	TWI%	WI	TWI%	WI	TWI%	WI	TWI%	WI	TWI%
A1	15.5	351	38.3	870	9.5	216	26.1	595	5.12	116	14.6	332	4.47	102	13.1	300
A2	12.1	275	29.9	680	7.43	169	20.5	465	4	91.1	11.4	260	3.5	79.1	10.3	233
A3	4.76	108	11.8	268	2.93	66.5	8.1	183	1.58	35.8	4.51	102	1.38	31.3	4.04	91.9
B1	10.8	246	26.8	608	6.64	151	18.3	416	3.58	81.4	10.2	232	3.12	71	9.18	209
B2/B3	4.3	97.7	10.6	242	2.64	60.4	7.28	165	1.42	32.3	4.07	92.4	1.24	28.3	3.65	83
D	3.14	71.3	7.77	177	1.93	43.8	5.31	121	1.04	23.6	2.97	67.4	0.91	20.6	2.66	60.6

## Data Availability

The original contributions presented in this study are included in the article/Supplementary Material. Further inquiries can be directed to the corresponding author.

## Supplementary Materials

The following supporting information can be downloaded at https://www.mdpi.com/article/10.3390/foods15101702/s1. Table S1. MS/MS parameters for PFAS analytes and internal standards. Table S2. Validation parameters for PFAS in eggs.
