# Supplementary material for: Home-Produced Eggs as Indicators of PFAS Contamination in Food Following a Fire at a Plastic Recycling Plant
Source: Foods. 2026 May 12;15(10):1702. doi: 10.3390/foods15101702 (PMC13206700; doi:10.3390/foods15101702)
Supplement: Supplementary file 1 [file foods-15-01702-s001.zip › foods-4290633-supplementary.pdf]

Table S1. MS/MS parameters for PFAS analytes and internal standards

Analyte	IS	Precursor ion *(m/z)*	Product ion *(m/z)*	Collision energy (eV)	Retention time (min)
**Perfluoroalkyl carboxylic acids (PFCA)**					
PFBA	$^{13}C_4$-PFBA	213	169	6	3.49
PFPeA	$^{13}C_5$-PFPeA	263	219	4	4.86
PFHxA	$^{13}C_5$-PFHxA	313	**269**	6	5.72
			119	22	
PFHpA	$^{13}C_4$-PFHpA	363	**319**	6	6.77
			169	18	
PFOA	$^{13}C_8$-PFOA	413	**369**	6	7.99
			169	18	
PFNA	$^{13}C_9$-PFNA	463	**419**	10	9.28
			219	18	
PFDA	$^{13}C_6$-PFDA	513	**469**	6	10.53
			269	18	
PFUnDA	$^{13}C_7$-PFUnDA	563	**519**	12	11.68
			269	16	
PFDoDA	$^{13}C_2$-PFDoDA	613	**569**	12	12.69
			319	22	
PFTrDA	$^{13}C_2$-PFDoDA	663	**619**	14	13.42
			169	34	
PFTeDA	$^{13}C_2$-PFTeDA	713	669	12	13.92
			**169**	32	
**Perfluoroalkyl sulfonic acids (PFSA)**					
PFBS	$^{13}C_3$-PFBS	299	99	44	5.01
			**80**	36	
PFPeS	$^{13}C_5$-PFHxA	349	99	36	5.82
			**80**	40	
PFHxS	$^{13}C_3$-PFHxS	399	119	36	6.82
			99	40	
			**80**	48	
PFHpS	$^{13}C_8$-PFOA	449	99	46	8.01
			**80**	50	
L-PFOS	$^{13}C_8$-PFOS	499	99	56	9.26
			**80**	56	
br-PFOS	$^{13}C_8$-PFOS	499	169	56	8.7-9.1 (2 peaks)
			99	56	
			**80**	56	
PFNS	$^{13}C_2$-8:2 FTS	549	99	48	10.49
			**80**	76	
PFDS	$^{13}C_7$-PFUnDA	599	99	60	11.61
			**80**	60	
PFUnDS	$^{13}C_2$-PFDoDA	649	**99**	55	12.61
			80	43	
PFDoDS	$^{13}C_2$-PFTeDA	699	99	60	13.34
			**80**	64	
PFTrDS	$^{13}C_2$-PFTeDA	749	**99**	59	13.87
			80	35	

The product ion (in bold) was used for compound quantitation.

Table S1. MS/MS parameters for PFAS analytes and internal standards (continued)

Analyte	IS	Precursor ion (*m/z*)	Product ion (*m/z*)	Collision energy (eV)	Retention time (min)
**Perfluoroalkane sulfonamides**					
FOSA	$^{13}C_8$-FOSA	498	478	21	11.82
			**78**	38	
**PFAS Substitutes**					
DONA	$^{13}C_3$-PFHxS	377	**251**	12	6.87
			85	36	
HFPO-DA (GenX)	M3HFPO-DA ($^{13}C_3$-HFPO-DA)	285	185	16	6.00
			**169**	4	
			119	30	
9Cl-PF3ONS	$^{13}C_2$-8:2 FTS	531	**351**	28	10.00
			99	28	
			83	32	
11Cl-PF3OUdS	$^{13}C_8$-FOSA	631	**451**	32	12.21
			83	32	
Capstone A	$^{13}C_9$-PFNA	527	**507**	7	9.71
			181	11	
Capstone B	$^{13}C_8$-PFOS	569	**549**	11	9.20
			223	13	
**Fluorotelomer sulfonates (FTS)**					
4:2 FTS	$^{13}C_2$-4:2 FTS	327	**307**	16	5.63
			81	44	
			80	32	
6:2 FTS	$^{13}C_2$-6:2 FTS	427	**407**	28	7.91
			81	44	
			80	44	
8:2 FTS	$^{13}C_2$-8:2 FTS	527	**507**	32	10.50
			80	52	
**Isotopically labelled standards (IS)**					
M3HFPO-DA ($^{13}C_3$-HFPO-DA)	-	287	185	20	6.00
**MPFAC-24ES**					
$^{13}C_4$-PFBA	-	217	172	5	3.49
$^{13}C_5$-PFPeA	-	268	223	5	4.86
$^{13}C_5$-PFHxA	-	318	273	10	5.72
$^{13}C_4$-PFHpA	-	367	322	7	6.77
$^{13}C_8$-PFOA	-	421	376	7	7.99
$^{13}C_9$-PFNA	-	472	427	7	9.28
$^{13}C_6$-PFDA	-	519	474	10	10.53
$^{13}C_7$-PFUnDA	-	570	525	10	11.68
$^{13}C_2$-PFDoDA	-	615	570	9	12.69
$^{13}C_2$-PFTeDA	-	715	670	11	13.93
$^{13}C_8$-FOSA	-	506	78	39	11.82
$^{13}C_3$-PFBS	-	302	80	38	5.00
$^{13}C_3$-PFHxS	-	402	99	39	6.82
$^{13}C_8$-PFOS	-	507	99	47	9.26
$^{13}C_2$-4:2 FTS	-	329	309	24	5.63
$^{13}C_2$-6:2 FTS	-	429	409	28	7.91
$^{13}C_2$-8:2 FTS	-	529	509	28	10.50

The product ion (in bold) was used for compound quantitation.

Table S2. Validation parameters for PFAS in eggs.

Analyte	LOQ ($\mu g\ kg^{-1}$)	Apparent recovery (%)	Relative standard deviation (%RSD_R)	Relative expanded uncertainty (k = 2)
**Perfluoroalkyl carboxylic acids (PFCA)**				
PFHxA	0.025	103.4%	9.3%	25.3%
PFHpA	0.025	106.2%	7.9%	23.4%
PFOA	0.05	104.3%	11.0%	28.4%
PFNA	0.025	106.4%	5.6%	22.7%
PFDA	0.025	114.1%	14.5%	22.7%
PFUnDA	0.05	99.9%	10.4%	16.6%
PFDoDA	0.025	105.7%	21.7%	31.9%
PFTrDA	0.10	97.9%	16.6%	46.8%
PFTeDA	0.15	109.7%	15.7%	62.4%
**Perfluoroalkyl sulfonic acids (PFSA)**				
PFBS	0.025	104.2%	8.9%	26.0%
PFPeS	0.025	99.1%	8.0%	25.8%
PFHxS	0.025	109.9%	9.2%	25.1%
PFHpS	0.025	94.1%	8.3%	32.9%
L-PFOS	0.020	111.7%	9.9%	27.8%
br-PFOS	0.10	104.0%	17.4%	30.5%
PFNS	0.05	86.0%	23.2%	31.4%
PFDS	0.025	126.2%	17.9%	31.4%
PFUnDS	0.10	76.7%	16.9%	46.8%
PFDoDS	0.10	102.3%	18.1%	31.7%
PFTrDS	0.15	72.0%	12.3%	48.8%
**Perfluoroalkane sulfonamides**				
FOSA	0.025	104.3%	9.1%	28.5%
**PFAS Substitutes**				
DONA	0.05	93.9%	18.7%	36.9%
HFPO-DA (Gen X)	0.025	104.3%	11.8%	32.8%
9Cl-PF3ONS	0.05	96.8%	15.8%	30.7%
11Cl-PF3OUdS	0.025	128.0%	17.2%	17.6%
Capstone A	0.025	101.4%	24.8%	48.7%
Capstone B	0.05	93.6%	23.9%	45.5%
**Fluoroteleomer sulfonates (FTS)**				
4:2 FTS	0.05	88.8%	16.6%	25.4%
6:2 FTS	0.05	88.9%	16.6%	24.7%
8:2 FTS	0.05	88.8%	18.7%	32.1%
